# Environmental and occupational respiratory diseases – 1047. Trend of asthma and attention deficit – a 5-year population survey

**DOI:** 10.1186/1939-4551-6-S1-P46

**Published:** 2013-04-23

**Authors:** Kuo-Wei Yeh, Jing-Long Huang

**Affiliations:** 1 Division of Pediatric Allergy Asthma and Rheumatology, Department of Pediatrics, Chang Gung Memorial Hospital, Taoyuan, Taiwan; 2 Department of Pediatrics, Chang Gung Memoiral Hospital, Taipei, Taiwan

## Background

The aim of this study was to investigate the incidence and trend of comorbid conditions of attention deficit developed among asthma patients during 5 years period.

## Methods

This study was based on continuous data from the Taiwanese National Health Insurance Research Database which contained 98% of registry files of all 22.60 million populations. Asthma was selected with code 493.xx of the International Classification of Disease, 9th Revision, and Clinical Modification. Asthma case was defined that the diagnosis occurred more than three times within 6 months. A total 139,629 (74,603 in male, 65,026 in female) patients newly diagnosed with asthma in 2003 were enrolled in this cohort and follow-up. Attention deficit was defined as patients with first diagnosis ICD-9 code of 314.xx.

## Results

There were 0.3% of asthma patients suffering from attention deficit in year 2003 and increased to 1.0% in year 2008. The male asthma patients suffered from attention deficit increased from 0.46% to 1.69% in, which is more common than females (from 0.12 to 0.40, p<0.001). The male asthma patients had 4-fold risk than females to have this problem. Comparing to non-asthma populations, both genders asthma patients presented with almost equivalent risk, though statistical significantly, to have attention deficit (p<0.001).

## Conclusions

Asthma patients significantly increased to develop attention deficit, and male patients had increasing trend within 5 years follow-up period.

